# Modifiable reporting unit problems and time series of long-term human activity

**DOI:** 10.1098/rstb.2019.0726

**Published:** 2020-11-30

**Authors:** A. Bevan, E. R. Crema

**Affiliations:** 1 Institute of Archaeology, University College London, London WC1H 0PY, UK; 2 Department of Archaeology, University of Cambridge, Downing Street, Cambridge CB2 3DZ, UK

**Keywords:** archaeology, radiocarbon, settlement, population, research bias

## Abstract

This paper responds to a resurgence of interest in constructing long-term time proxies of human activity, especially but not limited to models of population change over the Pleistocene and/or Holocene. While very much agreeing with the need for this increased attention, we emphasize three important issues that can all be thought of as modifiable reporting unit problems: the impact of (i) archaeological periodization, (ii) uneven event durations and (iii) geographical nucleation-dispersal phenomena. Drawing inspiration from real-world examples from prehistoric Britain, Greece and Japan, we explore their consequences and possible mitigation via a reproducible set of tactical simulations.

This article is part of the theme issue ‘Cross-disciplinary approaches to prehistoric demography’.

## 1. Introduction

**Figure 1.**
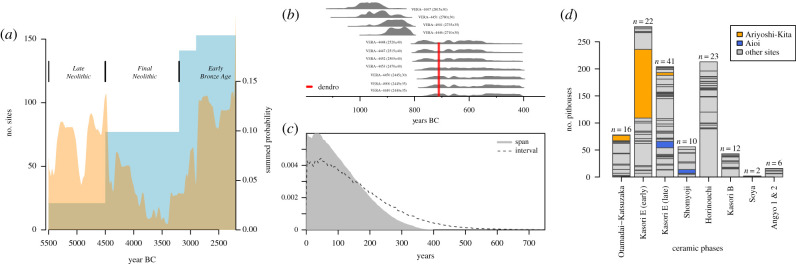
Motivating examples from (*a*) prehistoric site counts and summed radiocarbon from the Peloponnese, Greece [11]; (*b,c*) individual radiocarbon dates, dendrochronological dates and a duration model from the ‘Mauk E’ Bronze Age copper mine, Austria [12,13]; and (*d*) counts of Middle and Late Jomon period pit-dwellings and settlements in Eastern Tokyo Bay [3]. (Online version in colour.)

The last few years have seen a real resurgence of interest in how best to construct long-term time proxies of human activity, whether with regard to changes in the aggregate human population (e.g. this journal issue; [1,2]), settlement patterns [3–6], land cover and land use [7,8], metal production and deposition [9] or food storage strategies [10], to name but a few. This fresh ambition for a systematic longitudinal view goes well beyond the traditional construction of archaeological typologies or chronologies for their own sake and looks to contribute more meaningfully to wider, cross-disciplinary, *longue durée* debates about change over the Pleistocene and Holocene. It is also healthy in highlighting all sorts of methodological challenges that have been lurking in the shadows of archaeological practice for far too long. In this paper, we wish to focus on three particularly salient issues that in different ways can all be thought of as *modifiable reporting unit problems*: the impact of (i) archaeological periodization, (ii) uneven event durations and (iii) geographical nucleation-dispersal phenomena. Examples from the archaeology of prehistoric Britain, Greece and Japan are introduced as substantive real-world motivations (figure 1), accompanied by ‘tactical’ simulations (see [14]) that explore both the likely consequences of these issues and their possible mitigation. Details about the simulations can be found in the electronic supplementary material, and R scripts and data for generating all the figures are available as a dedicated repository (https://github.com/ercrema/repunitprobs, doi:10.5281/zenodo.3839249).

## 2. Periodization effects

Since the earliest days of archaeology as a formal subject, practitioners have sought to classify past human culture in both space and time, for instance by finding pottery types that come from the same region and belong to the same chronological horizon (a desire to lump-or-split continuous variation in scientific observations that is of course not restricted to archaeology: see Womble [15]; also archaeological discussion in Lucas [16] and Perreault [17, pp. 23–39]). These culture-historical pigeonholes still offer key building blocks for our relative dating schemes and often become implicit narrative protagonists in our stories about the past (e.g. ‘the rise, expansion and decline of the corded pottery-making people’, to stereotype slightly: see [18] for a good discussion). We certainly do not wish to argue below that such efforts to assign culture to periods are now somehow wholly misjudged or outdated, but we do want to emphasize that they have unintended interpretive and analytical consequences, especially when we look to *count up, correlate or otherwise compare quantitatively* various kinds of evidence for human activity through time (e.g. lithics, pots, sites, houses, etc.), using these periods as modifiable reporting units. For example, in Greece, an increasingly discussed chronological and cultural problem occurs at the transition from the Neolithic to the Bronze Age [11,19], where despite a concerted effort to sample potential candidate sites, very few radiocarbon dates fall in a so-called ‘missing millennium’ at roughly 4000–3000 BCE (figure 1*a*). While the settlement patterns in northern Greece largely match this perceived drop in radiocarbon dates (not shown here, see [11]), those in southern Greece look more complicated with what at first glance appears to be a consistent rise in site counts from later Neolithic to Final Neolithic to Early Bronze Age (figure 1*a*). However, comparison of summed radiocarbon, southern Greek site counts and associated period boundaries suggests that at least part of the discrepancy between them may simply be about where ‘Final Neolithic’ stops and starts as a counting unit (hence a periodization problem), while a further complicating issue is certainly also the move from likely longer lasting, more nucleated earlier Neolithic sites to a more dispersed later settlement of likely shorter duration (see §§3 and 4).

**Figure 2.**
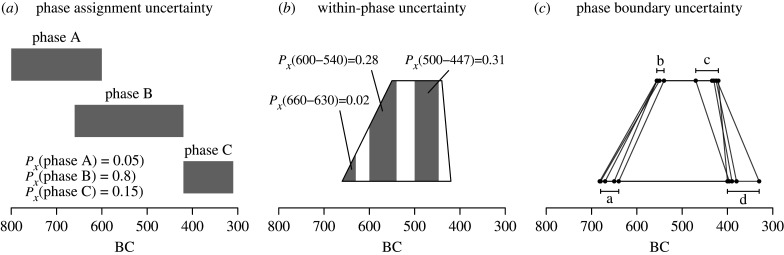
Different forms of chronological uncertainty in archaeological periodization: (*a*) event x is assigned to three possible archaeological phases, with phase B showing the highest probability; (*b*) the uncertainty within the phase is defined by a trapezium distribution, from which probability of occurrence for particular time intervals can be derived; (*c*), five possible shapes of the trapezium determined by the uncertainty in the definition of its parameters a, b, c and d.

In any case, archaeological periodization introduces a host of further related problems that are certainly worth addressing in more detail. The first form of uncertainty (following [20]) is to do with how confidently we can assign any event to a particular phase or period (*phase-assignment uncertainty*), which typically arises from limitations in the quality and quantity of culturally diagnostic elements (how recognizable the relevant pottery, lithic or other chronological indicators are in one period relative to another period). For example, Roman pottery may be easier for archaeologists in a particular region to identify than Late Bronze Age pottery, regardless of whether the amount of activity in these two periods was in fact similar. As a result, the count of Roman pots and Late Bronze Age pots are difficult to compare equitably (e.g. [21]). Furthermore, even if we are confident that an event belongs to a certain period, it is rarely if ever clear what shape of probability best expresses the likelihood of its occurrence within that period (*within-phase uncertainty*). For example, if a particular episode of house construction *x* belongs to a period dated between 600 and 300 BCE, what is *p_x_*(*t* = 340), the probability that house was built in 340 BCE? This form of uncertainty is generally quantified by a fairly arbitrary choice of some probability distribution that describes how *p*(*t*) changes for any value (e.g. calendar year) of *t*. The example shown in figure 2*c* is a ‘trapezium’ distribution (see also [22]), but elsewhere a flat, uniform distribution is often assumed (the application of aoristic analysis in archaeology, e.g. [23–25], etc.). Finally, the boundaries of a known period (e.g. respectively, 600 and 300 BCE) are themselves parameters that are imprecisely dated approximations of more complex cultural changes and hence have their own uncertainties (*phase boundary uncertainty*). We do not really know exactly when the Late Bronze Age stops and starts down to the precise year (and would typically laugh at the idea that such precision was appropriate for what most consider an archaeological ‘ballpark’ estimate). Figure 2 tries to capture the essence of the three kinds of uncertainty mentioned above for any hypothetical event *x*.

While there is no single perfect solution for quantifying these uncertainties, a growing number of studies now employ some combination of probabilistic estimates and Monte Carlo approaches to generate simulation envelopes to define how these uncertainties affect what conclusions we can or cannot reliably draw from particular time series (e.g. [3,20,26,27]). At a smaller scale, Monte Carlo Markov chain approaches can also be useful for combining ‘hard’ (e.g. multiple absolute dates) and ‘soft’ (e.g. stratigraphic relationships) into a final probabilistic model of a particular sequence [28].

**Figure 3.**
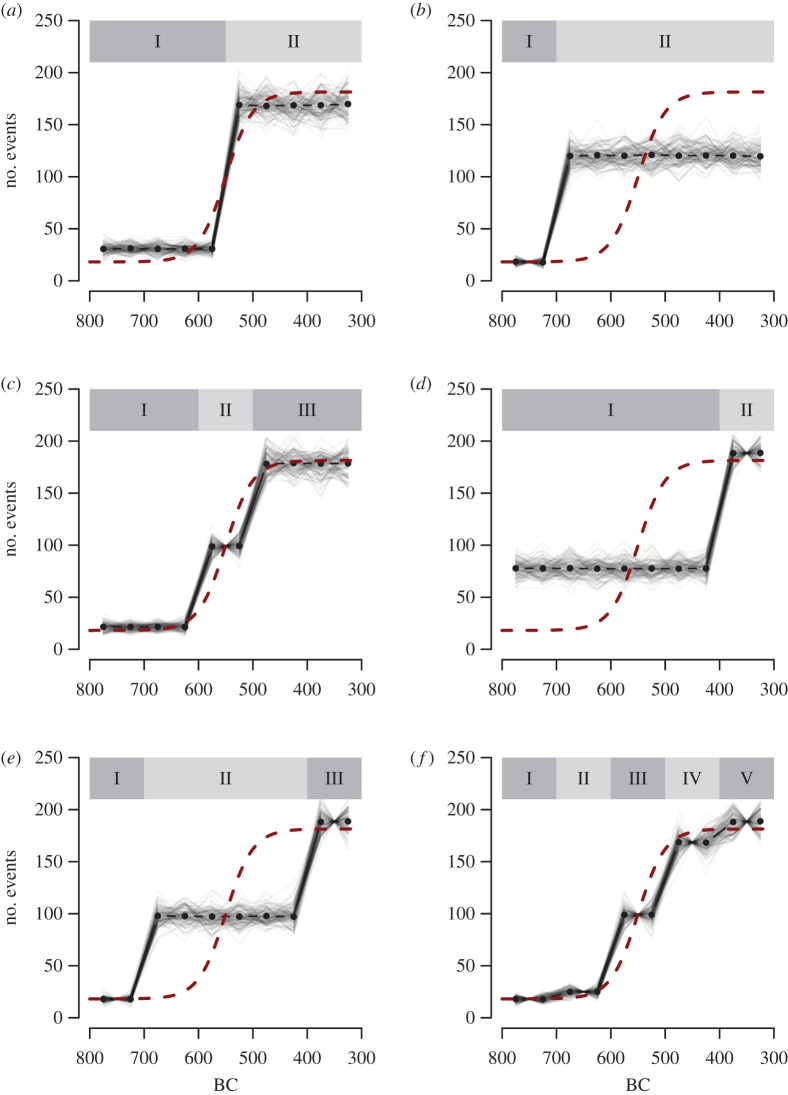
The effect of modifiable temporal unit problem on simulation-based approaches for handling chronological uncertainty and archaeological periodization. The dashed line in each panel indicates the ‘true’ population dynamic based on a count set of hypothetical events such as house constructions, with a logistic growth occurring between 600 and 500 BC. A sample of 1000 events generated from the population curve was aggregated and assigned to different archaeological periods (with their temporal demarcation shown at the top of each panel). This was then used to carry out a Monte Carlo analysis where the time-stamp of each event was simulated (assuming a uniform distribution within each phase) and then aggregated into 50-year time blocks. This process was repeated 1000 times so that a simulation envelope can be generated for each scenario as well as average estimates shown as solid dots and a line. Further details of this procedure and the associated R script can be found in the electronic supplementary material. (Online version in colour.)

Even if we could manage all of the above uncertainties well, there would still be a major issue arising from the fact that archaeological periodizations are *always arbitrary slicings of the temporal dimension*, just as any political, ethnic or linguistic borders that are drawn on a map are invariably arbitrary slicings of the spatial dimension too. The spatial analogy is apt because we are referring here to the temporal equivalent of a *modifiable areal unit problem* identified long ago by geographers (e.g. [29,30]). In what follows, we therefore refer to this problem of cultural periods as a *modifiable temporal unit problem.* Figure 3 illustrates the main point via a tactical simulation. Assume we have a time series representing population change over a 500-year period of prehistory, and that the shape of this hypothetical population trend is logistic (as indicated by the dashed red line, implying initial slow growth, then fast growth, then a population plateau often assumed to imply the hitting of a local carrying capacity). We can sample at random 1000 events from this logistic distribution (e.g. newly built houses or individual human burials) and then convert their absolute calendar ages (year of birth or death) to a nominal-scale label referring to its assigned phase.

Figure 3 explores the repercussions of this for different numbers of periods and different period boundaries: thus a house built in 630 BC would be affiliated with phase I in figure 3*a*, but with phase II in figure 3*e*. The simulation can be repeated many times (see electronic supplementary material, for further details). And the results reveal how, even if we only consider *within-phase uncertainty* (in this case assuming a uniform probability distribution) the resulting time series would look rather different depending on how archaeological periodizations slice time, leading potentially to biased estimates about when the major population growth occurred. If either the timing of the population change corresponds closely to a transition from one archaeological period to another (figure 3*a,c*) or if the number of archaeological periods is sufficiently high to ensure a detailed chronological resolution (figure 3*f*), then the bias introduced by archaeological periodization is comparatively small. However, if there is a mismatch between the onset of these population changes in the time series and the way the time series evidence is segmented into periods, then there could be a far greater error in estimates of the timing and rate of change. To be fair, we might anticipate that a major population shift will often correspond with the kind of major cultural transition behind the definition of an archaeological period, but of course this correspondence should be independently demonstrated in each case, not simply assumed to hold true. In real-world settings, the problem can further be exacerbated by *phase-assignment uncertainty* (how confidently any event be assigned to a particular period/phase, see above) and typically an archaeologically recovered sample of dwellings (or other counted features) will not be identical to the deliberately idealized model in figure 3, but instead will be dated with varying certainty (so some houses will be confidently assigned to one period, some merely attributely to a broader span across several periods).

How best to address this challenge? One option is to use a more nuanced and tailored model with which to represent *within-phase uncertainty*. The mismatch between the true population curve and the time series derived from data aggregated into periods is exacerbated by how the within-phase chronological uncertainty is modelled. In the case of figure 3, as well as in the application of aoristic analysis and similar methods, the within-phase uncertainty is modelled by a uniform probability distribution. Thus, for example, an event associated with phase II in figure 3*e* is assumed to have the same chance of being from the interval 650–600 BC and the interval 500–450 BC while in reality it is far more likely that the event is from the latter temporal bracket. An appropriate model that captures correctly the within-phase uncertainty would thus overcome the modifiable temporal unit problem, but this would require unusually high chronological precision. Analysing the frequency of residential data would, for example, ideally require sufficiently large samples of radiocarbon dates associated with dwellings that are assigned to the focal period. Of course if there is such a dataset available there would be no need to find a statistical solution in the first place. Another would be to reshape our relative artefact chronologies to produce probability distributions per calendar year (e.g. based on geomorphometric distances, Jaccard distances of trait similarities, co-occurrence seriation, etc.) but again this is a very significant undertaking and not a strategy for working with the mass of archaeological evidence that we already have in legacy form (e.g. [31]). Another approach therefore consists of testing whether observed time series are different from theoretical models, by emulating the information loss and bias introduced by archaeological periodization. This is effectively the same principle behind approaches designed to determine whether empirical summed radiocarbon probability distributions (SPDs) deviate from theoretical growth models (see [1,32], etc.; see also for possible lateral offset effects in radiocarbon dates, [33]). In this case, the choice of suitable growth model and associated parameters might be more difficult to retrieve from the observed data, and the statistical power of the analysis is likely to be conditioned by both the null model and by the archaeological periodization. Nonetheless, if explicit hypotheses are available, this approach could potentially overcome the modifiable temporal unit problem.

## 3. Duration effects

Many archaeological phenomena are not discrete events, but instead exhibit durations over time. A house can last for 1 year or for 100 before it is abandoned. A ‘settlement’ is a convenient, but also ambiguous, term referring to multiple dwellings (i.e. a modifiable reporting unit). Settlements can vary considerably, not only in size but also in duration: they can last for six months (e.g. the recently discovered Bronze Age pile-dwelling site of Must Farm, south-eastern Britain: [34] or for thousands of years (e.g. a Middle Eastern tell site such as Aleppo, north-western Syria), with very different consequences for the resulting mixes or palimpsests in our evidence (e.g. [17,35, pp. 41–48]; one reason why we often try to discern site sub-phases where mixing is considered less troublesome). We also frequently create further problems by conflating the chronological uncertainty we have about past events with the expected duration in the past: in other words, if we can only date a site to 1000–400 BCE based on observable finds, we often make interpretations that assume that the site was continuously occupied over this period. A good example of how misleading this might be is offered by the Late Bronze Age copper mine at Mauk E in the Austrian Tyrol (figure 1*a*; [12,13]). This mine produced some radiocarbon dates that likely exhibit ‘old wood’ effects, but even excluding these, the rest of the sample visually suggests a duration of many centuries, due to a combination of the accompanying ^14^C measurement uncertainty and a plateau in the calibration curve at this point (the well-known ‘Hallstatt plateau’). Bayesian modelling of the likely duration improves things if we assume the dates are representative of the total activity at the site (figure 1*b*), but not if we simply model the difference between likely start and end dates (a distinction in OxCal software between a ‘span’ and an ‘interval’ model, see [28]). In fact, tree-ring sequences from various timber supports at Mauk E suggest an even shorter likely duration of about at most a few decades (900–869 BCE).

**Figure 4.**
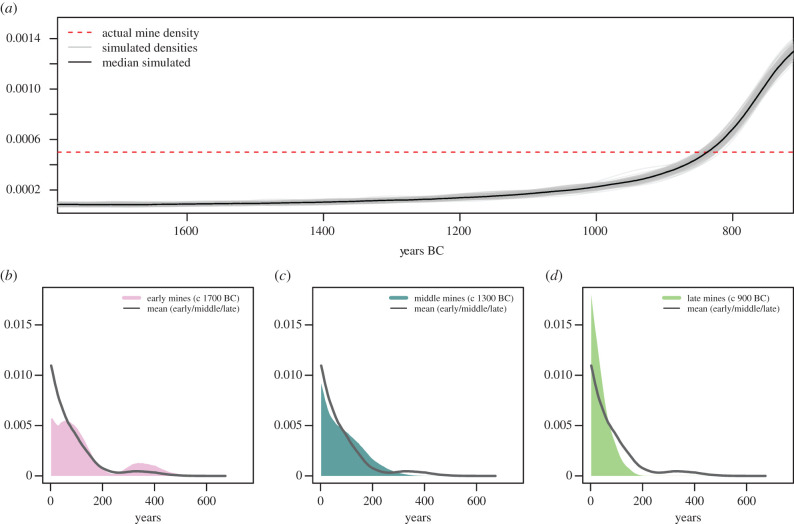
A simulation of copper mines over one millennium: (*a*) the discrepancy between a constant number of in-use mines as defined by the model setup, and the misleading count of mines that arises if mine duration is ignored or unknown, (*b–d*) pooled OxCal span probabilities for five mines each from early, middle and late parts of the 1750–750 BCE time span, each dated via five hypothetical radiocarbon dates. (Online version in colour.)

We can further use copper mines as a conceptual prompt for another tactical simulation. Imagine a simple example in which, for each year over a millennium time span from 1750 to 750 BCE, there are always exactly 100 copper mines of equal size each year that are producing copper for Bronze Age Europe. The only thing we alter is the duration of each copper mine and how certain we are about this duration. We model a gradual linear change in average mine use duration from 200 years on average in 1750 BCE to 10 years 750 BCE on average (perhaps due to changing water table conditions, erosion, available people to mine, political circumstance, quality of ore body, etc.). Whenever an old mine is abandoned and falls out of use, a new mine is setup with a use-life (aka duration) drawn at random from a negative binomial distribution whose mean declines through the period of interest as described above. Such a choice is appropriate given a negative binomial distribution is frequently used to model waiting times until a failure (in this case mine abandonment). Figure 4*a* juxtaposes a correct, uniform pattern of unchanging mine counts through time (red dashed line), with the dramatically different pattern observed if all mines are assumed to be of similar duration. The potential risk for misinterpretation is hopefully obvious. On a more positive note, if sufficient absolute dating evidence exists, the relevant signal for this problem should be retrievable. For example, if we take just 15 mines spread out across early, middle and late parts of the sequence and sample five hypothetical radiocarbon dates at each mine (a plausible, financially viable amount of radiocarbon sampling; see electronic supplementary material, for sampling routine, back-calibration and error modelling), an OxCal span model does correctly indicate this likely changing trend, allowing us perhaps to adjust our modelling and interpretation accordingly. While similar issues have been explored in the past, for instance in relation to archaeological periodization and the contemporaneity problem (e.g. [36,37], more recently [38]), the implications of duration have been underexplored in methodological applications such as aoristic analysis or summed radiocarbon (but see [24], appendix A; [6]).

## 4. Aggregation effects and spatially dependent sampling

As the above has already made clear, one lurking and well-known issue with any count-based population proxy is the question of whether an extra unit of the proxy straightforwardly implies an extra unit of inferred activity or population (see [4] for a relevant recent review). In other words, can a doubling of the proxy—whether this is the count of excavated dwellings, of known sites or of radiocarbon dates, for instance—be straightforwardly interpreted as a doubling of population? In fact, rarely is this an easy assumption to sustain: such time series usually cover large periods of time in which there are changes in residential mobility, the degree of settlement nucleation or in energy expenditure [39]. A ‘settlement’ can thus have a changing meaning, sometimes referring to large cities while at others referring only to hamlets or farmsteads and yet potentially counting them all as equivalent (researchers working in settings where settlement seasonality is likely will already be attuned to such challenges over short time scales). Even individual dwellings cannot always be treated as equivalents over longer time periods, in the case for example where they do not maintain consistent numbers of co-residents (e.g. changes from or towards nuclear versus extended families). One commonly used solution to these problems is to apply a ‘weight’ to each reporting unit (e.g. using house floor area or settlement size, or more direct models of area-to-population ratio based on ethnographic data (e.g. [40,41]), but it often remains difficult to know how one would define such a weighing scheme in a reliable way across time, space and a very patchy archaeological record.

Spatial nucleation and dispersal also creates recovery bias: for example, dwellings are rarely, if ever, sampled independently of each other but instead are often ‘discovered’ (i.e. excavated or surveyed) in clusters. Figure 1*d* is an example from prehistoric Japan that illustrates this problem. The stacked bar chart shows the total number of Jōmon pit-dwellings from Eastern Tokyo Bay [3] organized into chrono-typological periods and divided by archaeological sites. Some periods (e.g. Early Kasori E) are characterized by a skewed size distribution, with few sites (such as Ariyoshi-Kita) having a large share of the total number of residential units, while other periods (e.g. Late Kasori E) have a more even distribution of settlement sizes. It follows that the exclusion of some sites (e.g. Aioi) from the sample has a small impact on the time series of pit-dwelling counts, while the removal of others (e.g. Ariyoshi-Kita) can have a drastic impact.

**Figure 5.**
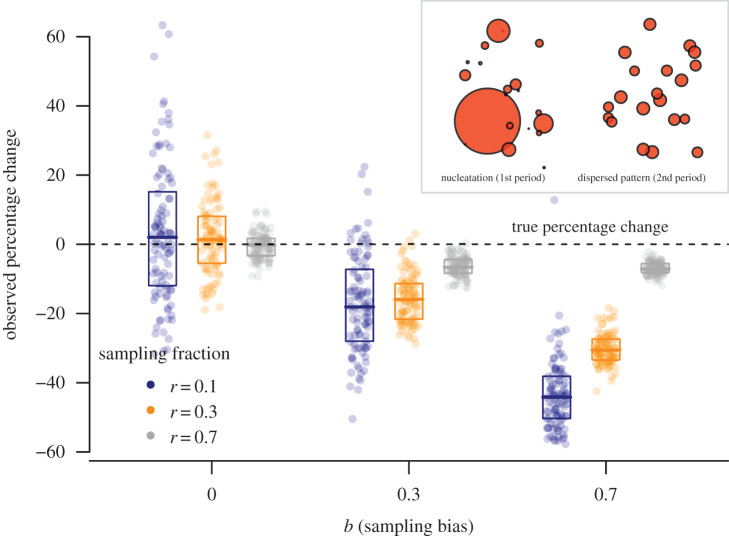
False signatures of decline in the number of residential units in a simulated dataset with different levels of sampling fraction (*r*) and site size-based sampling bias (*b*). (Online version in colour.)

To further illustrate this issue, we employ another tactical simulation (figure 5, see electronic supplementary material, for further details) in which the simulated data consist of two archaeological periods with the same duration in time and the same number of residential units, but different settlement sizes. In the first period, there are a few large nucleated settlements but many smaller sites. In the second period, settlements are in contrast mostly of similar size (see inset in figure 5). Because the number of residential units is the same in both periods, the ‘true’ percentage change between the two periods is 0 (total population is the same, only the spatial structure of that population across the landscape has changed). The simulation then emulates typical archaeological excavation or survey procedures in sampling different fractions of the total (in this case 0.1, 0.3, and 0.7), with a size-dependent detection probability defined by the parameter *b*. When the latter is set to 0, all sites (and consequently residential units within them) have the same probability of being sampled. When *b* > 0, larger sites (i.e. those with a larger number of residential units) have a higher chance of being included in the sample, and when *b* = 1 the probability of a site being detected is directly proportional to the relative contribution of its total number of residential units to the total number of dwellings for both periods. The results show that when sampling is unbiased (i.e. when *b* = 0 and all settlements have the same chance of being selected), the average percentage change of residential frequency across the simulations converge to the true value (i.e. 0%), with smaller sampling fraction showing more variability in the outcome as a consequence of sampling error. However, in the presence of size-based sampling bias, the average rate of change across the 100 simulations becomes negative, incorrectly suggesting a decline in the number of residential units between the two periods. The magnitude of this false signature is greater for smaller samples and/or stronger sampling bias. A similar interplay between sample size, sample error and the spatial patterning of the archaeological record has also been discussed extensively in the literature on field survey (e.g. [42]).

This exercise highlights an important interplay between how our population proxies are distributed across space and how they are sampled. These two factors cannot be assumed to be constant over time and ignoring them can lead to major misinterpretations of the available evidence. Summed radiocarbon date distributions are another approach where such nucleation–dispersal issues raise problems. Sampling for radiocarbon varies considerably from site to site: many radiocarbon dates might be taken by a wealthy research project at one site, while just one or two dates might be taken at another site of similar size (e.g. one excavated under rescue conditions). Put another way, large settlements are not always conveniently associated with a large number of radiocarbon dates and small settlements with a small number. To overcome this problem, Shennan *et al*. [1] introduced the idea of aggregating radiocarbon dates that are ‘close’ in time from the same site where this ‘binning’ procedure is effectively a trade-off which reduces the impact of uneven sampling for radiocarbon dates, but at the cost of treating all sites or phases as of equal effective size (see also [32]).

Responding to this issue, Crema & Kobayashi [20] demonstrated general agreement between a times series of summed radiocarbon dates where within-site dates are binned and a time series of dwelling counts, but they also revealed significant short-term discrepancies in the rate of change, at likely moments where prehistoric Japanese settlement patterns switched from more nucleated to more dispersed patterns (or vice versa). Downey *et al*. [43] proposed one possible solution by using the ‘community size’ variable in the Standard Cross-Cultural Sample [44] to infer a scaling factor by which to adjust the contribution of binned dates for European Mesolithic versus Neolithic populations. Ahn & Hwang [45] tried a different approach and only considered radiocarbon dates associated with individual residential units in the Korean peninsula effectively making the resulting SPD a proxy for residential density. Because all samples were related to a particular type of event (the use of a dwelling structure) rather than an ensemble, they were able to combine multiple radiocarbon dates from any given individual residential unit (via Bayesian rules and the *R_Combine* function in OxCal). Although we are not aware of other studies employing these specific approaches to improve the construction of SPDs, they represent a useful basis for developing more refined ways to handle radiocarbon dates as demographic proxies, for example by employing scaling factors such as floor area to population size [40,46]. That said, neither of the above two solutions is immune to further problems. The scaling solution adopted by Downey *et al.* [43] allows for larger samples, but it requires reliable criteria for classifying dates into different groups (in their case ‘Mesolithic’ versus ‘Neolithic’), and more crucially it assumes uniformity within each class. There is ample evidence that the latter in particular is an incorrect assumption—roughly contemporary settlements can and do vary in size across space—but more crucially settlement size distributions can fluctuate over time via nucleation–dispersal cycles (e.g. [3,47,48]). The main disadvantage of the targeted sample solution suggested by Ahn & Hwang [45] is that its strict selection criteria are likely to drastically reduce sample sizes for most radiocarbon databases, and it is still not immune to the problem illustrated in the tactical simulation described in figure 5.

## 5. Conclusion

This paper has foregrounded three topics that we consider to be under-discussed so far in archaeological analysis and also rarely mentioned when archaeological data is used in cross-disciplinary studies of human and environmental history. In some instances, the discussion above has already tried to point to ways in which such challenges might be better diagnosed and mitigated while, in others, it has largely only sounded an alarm without providing a solution. In any case, we would strongly argue that all three forms of modifiable reporting unit problem need to be given priority attention if we are to reconstruct more reliable long-term time series of human activity.

## Supplementary Material

Electronic Supplementary Material

## Data accessibility

Data and reproducible code for all of the analysis discussed in the paper can be found at https://github.com/ercrema/repunitprobs.
