# Supplementary material for: Modifiable reporting unit problems and time series of long-term human activity
Source: Philos Trans R Soc Lond B Biol Sci. 2020 Nov 30;376(1816):20190726. doi: 10.1098/rstb.2019.0726 (PMC7741099; doi:10.1098/rstb.2019.0726)
Supplement: Electronic Supplementary Material [file rstb20190726supp1.pdf]

Electronic Supplementary Material for:

Modifiable reporting unit problems and time series of long-term human activity

Andrew Bevan & Enrico Crema

01 April, 2020

NOTE

This ESM was rendered using custom R functions available on this GitHub repository:

https://github.com/ercrema/repunitprobs

Simulation 1: Modifiable Temporal Unit Problem

Consider the following logistic population dynamic, where a major population increase occurs between 600 and 500 BC.

```
age = seq(300,800,1)
asymptote = 0.9
sl = 0.04
midpoint = 550
p = 0.1+asymptote / (1 + exp((midpoint - age) * sl))
d=data.frame(yr=rev(age),p=p)
plot(d$yr,d$p,xlim=c(800,300),type='l',ylim=c(0,1),xlab="BC",ylab="",axes=FALSE)
axis(1,at=seq(800,300,-100))
mtext(side=2,"Population Size",line=1.1)
box()
```

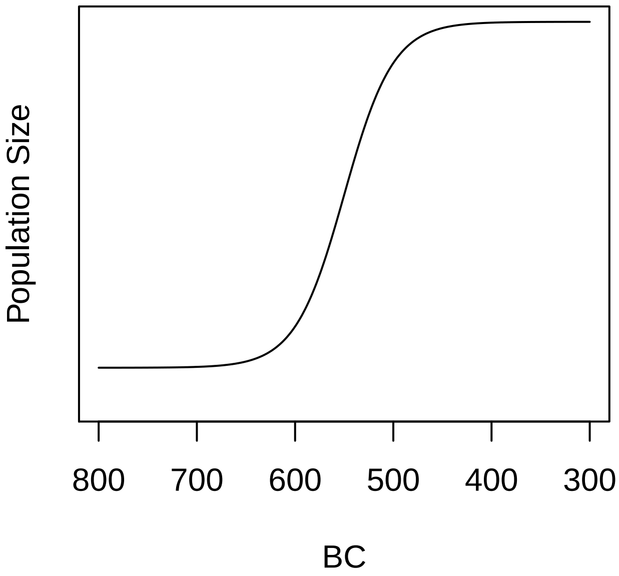

We first simulate 1,000 arhcaeological events that occured with frequencies directly proportional to such population dynamic. For example:

```
n=1000
set.seed(123)
ss = round(sample(d$yr,size=1000,prob=d$p/sum(d$p),replace=TRUE))
hist(ss,xlim=c(800,300),breaks=seq(300,800,50),xlab="BC",
     col='darkorange',border='lightgrey',freq=T,main='')
lines(d$yr,(d$p/(sum(d$p))*n*50),lwd=2,lty=2) #True Population Curve
```

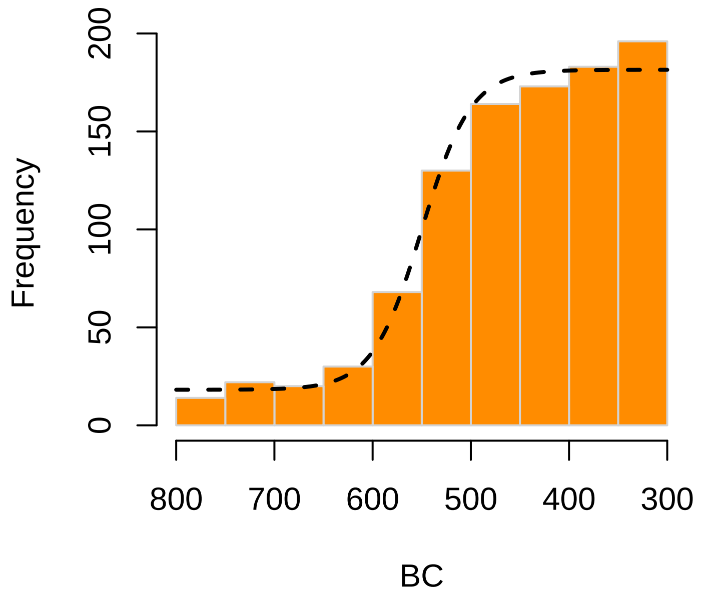

Although some discrepancy can be observed due to sampling error the observed density of sampled events and the underlying population are identical.

The Effect of Periodisation

We emulate an archaeological periodisation process by replacing the time-stamp of each event to the membership to an archaeological phase. For example, suppose phase A had a temporal span of 800 to 701, and phase B between 700 and 401, and phase C between 400 and 300.

```
  phases = cut(ss,breaks=c(800,700,400,300),labels=c("C","B","A"),include.lowest = TRUE)
#labels are in reverse order since dates are in BC
  table(phases)
```

```
## phases
##   C   B   A
## 379 585  36
```

Thus in this case there are 36 events assigned to phase A, 585 to phase B, and 379 to phase C.

Aoristic Analysis + Monte-Carlo Approach

There is a number of closely related developed in the last decade designed to analyse frequency data based on archaeological periodisation. One such approach consists of assigning probabilistic weights to individual events for a given temporal interval. This approach is at the basis of aoristic analysis, where time is divided into equally sized blocks, and weights are computed under the assumption of a uniform probability distribution within assigned phase(s). Thus if we use blocks of 50 years, we would obtain the following:

```
# Extract number of cases
An =table(phases)['A']
```

```r
Bn =table(phases)['B']
Cn =table(phases)['C']

# Create a weight matrix
weights = matrix(NA,nrow=n,ncol=10) #10 blocks of 50 years

# Assign weights
weights[1:An,] = rep(c(50/100,50/100,0,0,0,0,0,0,0,0),each=An)
weights[(An+1):(An+Bn),] = rep(c(0,0,50/300,50/300,50/300,50/300,50/300,50/300,0,0),each=Bn)
weights[(An+Bn+1):(An+Bn+Cn),] = rep(c(0,0,0,0,0,0,0,0,50/100,50/100),each=Cn)

# Compute Aoristic Sum
asum = apply(weights,2,sum)

# Visualise
midPoints = seq(775,325,-50)
plot(midPoints,asum,xlim=c(800,300),type='b',pch=20,xlab="BC",ylab="Aoristic Sum")
lines(d$yr,(d$p/sum(d$p)*n*50),lwd=2,lty=2,col='darkblue')
```

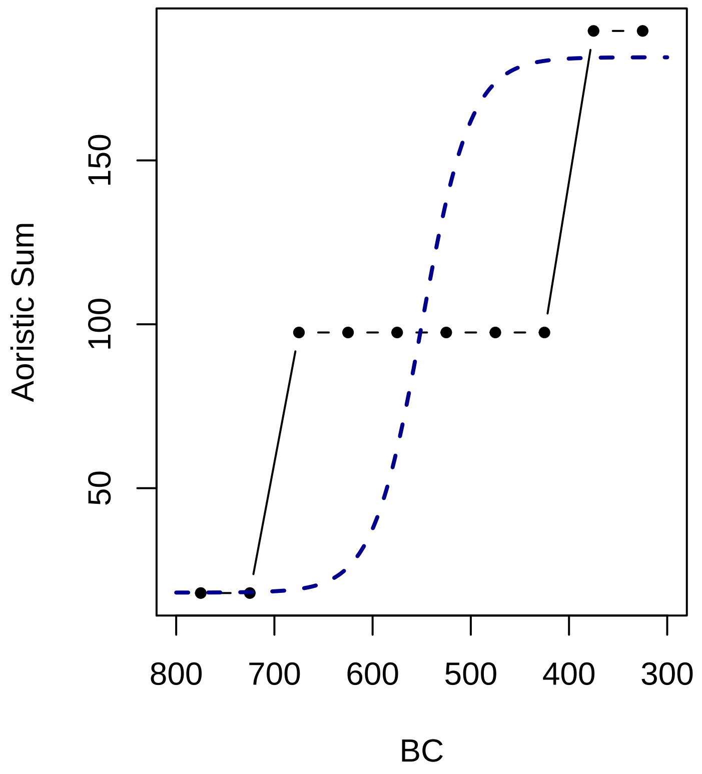

Aoristic sums do not provide, however, any measure of chronological uncertainty making hard to discern, for example, whether flat portions of the curve are the result of genuine stability in the frequency of events or the result of higher levels of uncertainty. To overcome this issue, and alternative approach consist of employing Monte-Carlo simulation:

```r
nsim =1000 #number of simulations
mat=matrix(NA,nrow=10,ncol=nsim)

# simulate dates and aggregate by 50 year blocks
for (s in 1:nsim)
{
  simdates = c(round(runif(An,min=701,800)),round(runif(Bn,401,700)),round(runif(Cn,300,400)))
  cnts = cut(simdates,breaks=seq(300,800,50),include.lowest = T)
  mat[,s]=as.numeric(rev(table(cnts))) # reverse order as dates are in BC
}

#make spaghetti plot with 100 random simulations
avg = apply(mat,1,mean)
plot(0,0,type='n',xlab='BC',ylab='Number of Events',xlim=c(800,300),ylim=range(mat))
apply(mat[,sample(1:1000,size=100)],2,lines,x=midPoints,col=rgb(0,0,0,0.05)) #
```

```
## NULL
```

```r
lines(midPoints,avg,type='b',pch=20)
lines(d$yr,(d$p/sum(d$p)*n*50),lwd=2,lty=2,col='darkblue')
```

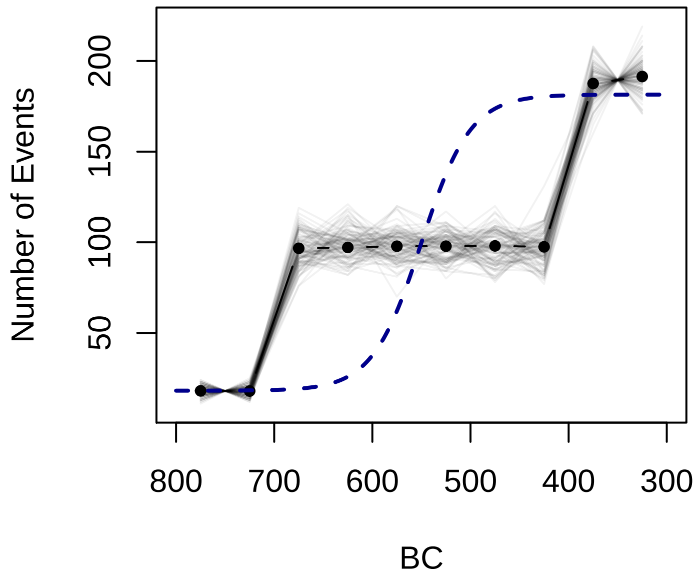

The average time-series extracted from the Monte-Carlo simulations is comparable to the result of the aoristic analysis, but showcases the extent of chronological uncertainty between 700 and 400 BC (i.e. phase B). More importantly, because of the assumption of uniform probability distribution, both methods fail to identify correctly the major population growth event between 600 and 500 BC. The extent of this bias depends on the resolution of the archaeological periodisation in relation to the scale of the population dynamic of interest and the extent by which shifts in frequencies co-occur with changes in archaeological phases.

The script below utilises the function `mcsim()` (which automatise the workflow described above) to generate different time-series with the Monte-Carlo method under different numnbers and position of archaeological

phases When the transition between archaeological periods are alligned with major shifts in the frequency of evens (e.g. panels *a* and *c*), or when the resolution of the phases are sufficiently fine-grained (e.g. panel *f*) the time-series obtained with the Monte-Carlo method recovers much of the features of the true population dynamics (shown in dashed red line). However when the resolution is coarse or when the shift from one archaeological period to another is not associated with changes in population density (e.g. panels *b*, *d* and *e*) the time-series will be biased in identifying the correct timing and magnitude of specific population dynamic:

```
resolution=50 #define time-block resolution
# Extract min-max from theorethical model
m=min((d$p/sum(d$p)*n*resolution))
M=max((d$p/sum(d$p)*n*resolution))


#Consider different periodisations:
LL = list(c(800,550,300),
          c(800,700,300),
          c(800,600,500,300),
          c(800,400,300),
          c(800,700,400,300),
          c(800,700,600,500,400,300))

par(mfrow=c(3,2),mar=c(5,5,1,1))
for (i in 1:length(LL))
{
  breaks=LL[[i]]
  tmp=mcsim(x=ss,nsim=1000,breaks=breaks,resolution=50)
  avg = apply(tmp,1,mean)
  plot(0,0,type='n',xlab='BC',ylab='Number of Events',
       xlim=c(800,300),ylim=c(0,250),axes=FALSE)
  axis(1)
  axis(2)
  apply(tmp[,sample(1:1000,size=100)],2,lines,x=midPoints,col=rgb(0,0,0,0.05)) #
  lines(midPoints,avg,type='b',pch=20)
  lines(d$yr,(d$p/sum(d$p)*n*50),lwd=2,lty=2,col='darkred')
  legend("bottomright",legend=letters[i],bty='n',cex=2)

   for (b in 1:(length(breaks)))
  {
    col='lightgrey'
    if (as.logical(b%%2)){col='darkgrey'}
    rect(xleft=breaks[b],xright=breaks[b+1],ybottom=210,ytop=250,border=NA,col=col)
    text(x=breaks[b+1]+(breaks[b]-breaks[b+1])/2,y=230,labels=as.roman(b))
  }

}
```

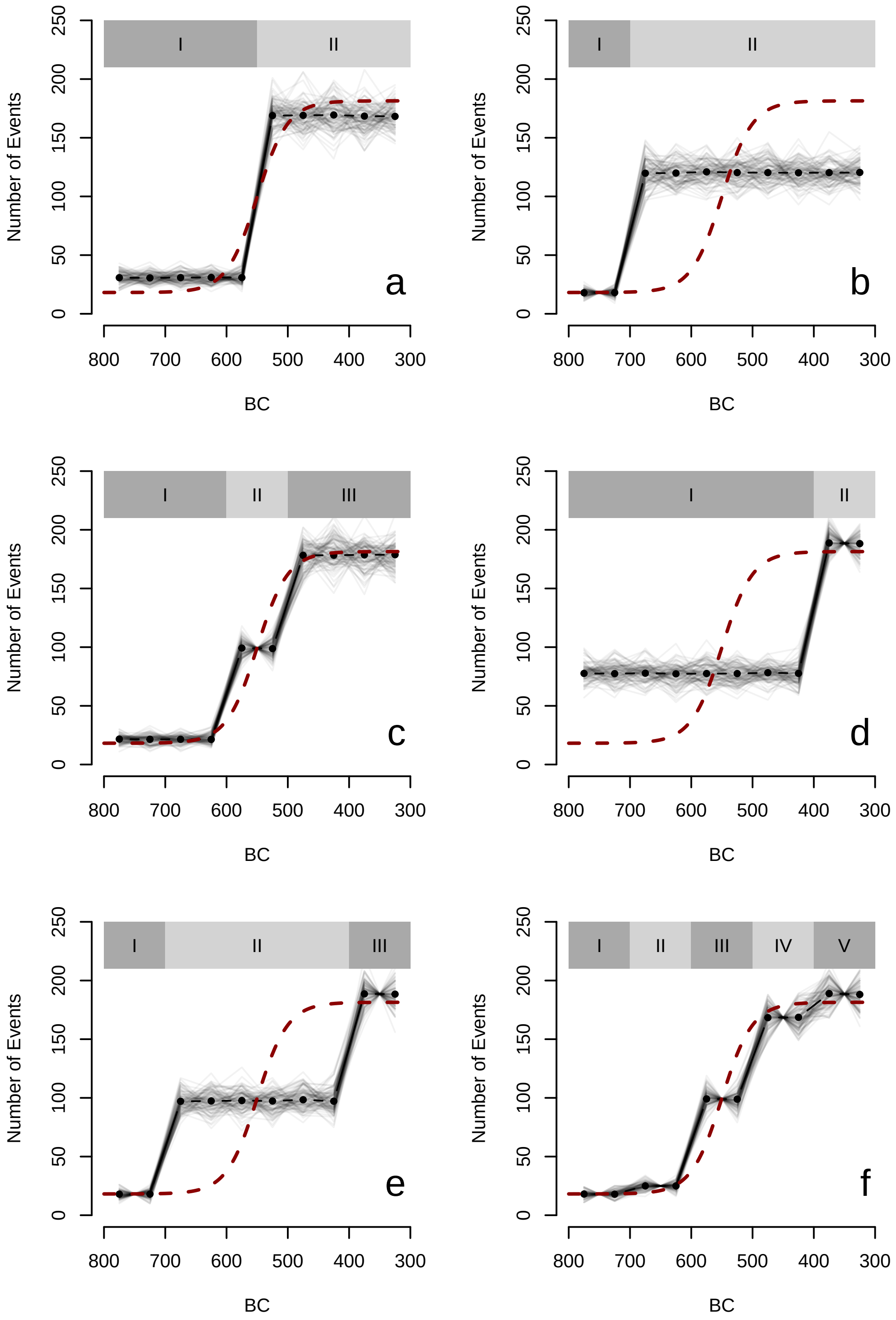
a
b
c
d
e
f
Number of Events
BC
I
II
III
IV
V
0
50
100
150
200
250
800
700
600
500
400
300

Simulation 2: Duration

Consider a time-series recording the number of simultaneously occupied mines over a 1,000 years interval (1750-750 BC). Suppose this number to be constant ($n = 100$) but with the duration of occupation of the mines to be a linear function of time. More formally we model the duration of each mine as a random draw from a negative binomial distribution with the dispersion parameter α equal to 1 and mean μ equal to

$$\mu(t) = -132.50 + -0.19t$$

with t between -1750 and -750. Here we limit μ to bet between 10 and 200 (i.e. if the result of the equation is below 10, μ is set to 10, if above 200, μ is set to 200). The R script below generates a 100 simulated dataset under these conditions:

```
## Parameters Setup
edgec <- 500 # Add +/-500 years to avoid edge effects
years <- c(-1750:-750) #Simulation Interval
simyears <- c((years[1]-edgec) : (years[length(years)]+edgec))
nmines <- 100 #Number of mines in a given yers
minepreallocation <- 10000

mDurationMax <- 200 #Maximum Duration of Sites
mDurationMin <- 10 #Minimum Duration of Sites
df <- data.frame(x=c(years[1],years[length(years)]),
                 y=c(mDurationMax,mDurationMin))
mod <- lm(y~x, data=df) #

## Simulate
usemodel <- TRUE
nsim <- 100
simmat <- matrix(nrow=length(simyears), ncol=nsim)
set.seed(100)


for (b in 1:nsim){
  minedf <- data.frame(StartBCE=c(rep(simyears[1],nmines),
                                  rep(NA,(minepreallocation-nmines))),
                       EndBCE=NA, Duration=NA, Mu=NA, Active=FALSE)
  minedf$Duration[!is.na(minedf$StartBCE)] <- rnbinom(nmines, mu=200, size=1)
  minedf$EndBCE <- minedf$StartBCE + minedf$Duration
  for (a in simyears){
    check1 <- minedf$StartBCE <= a & !is.na(minedf$StartBCE)
    check2 <- minedf$EndBCE >= a & !is.na(minedf$EndBCE)
    minedf[,"Active"] <- FALSE
    minedf[check1 & check2,"Active"] <- TRUE
    checksum1 <- 100-sum(minedf$Active)
    if (checksum1 > 0){
      myrows <- which(is.na(minedf$StartBCE))[1:checksum1]
      minedf$StartBCE[myrows] <- a
      if (usemodel){
        mu <- as.numeric(coefficients(mod)[1]) +
          (as.numeric(coefficients(mod)[2]) * a)
        if (mu > mDurationMax){
          mu <- mDurationMax
        } else if (mu < mDurationMin){
```

```
        mu <- mDurationMin
      }
    } else {
      mu <- 200
    }
    mu <- round(mu,0)
    minedf$Mu[myrows] <- mu
    minedf$Duration[myrows] <- rnbinom(checksum1, mu=mu, size=1)
    minedf$EndBCE[myrows] <- minedf$StartBCE[myrows] +
      minedf$Duration[myrows]
  }
}
minedf <- minedf[!is.na(minedf$StartBCE),]
nrow(minedf)
minedf$MidYear <- minedf$StartBCE+round(minedf$Duration/2,0)
tmp <- density(minedf$MidYear, n=length(simyears),
               from=simyears[1],to=simyears[length(simyears)])$y
simmat[,b] <- tmp
}
mediankd <- apply(simmat,1,median)
```

The figure below compares the life-span of a sample of 1000 sites from one simulation (top panel) along with $\mu(t)$ (dashed orange line), against the number of those occupied at a given moment in time (middle panel) and the frequency of mid points (lower panel).

```
set.seed(121)
par(mfrow=c(3,1),mar=c(0,4,3,4))
plotSUB=minedf[sample(1:nrow(minedf),size=1000),]
plot(0,0,type='n',xlim=c(-1800,-700),ylim=c(0,nrow(plotSUB)+1),
     axes=FALSE,ylab='',xlab='Year BC')
for (i in 1:nrow(plotSUB))
{
  lines(x=c(plotSUB$StartBCE[i],plotSUB$EndBCE[i]),y=c(i,i),lwd=0.5)
  points(x=c(plotSUB$StartBCE[i],plotSUB$EndBCE[i]),y=c(i,i),pch=20,cex=0.4)
  points(x=plotSUB$MidYear[i],y=i,pch=20,col='red',cex=0.6)
}
par(new=TRUE)
plot(0,0,type='n',xlim=c(-1800,-700),ylim=c(10,200),
     axes=FALSE,ylab='',xlab='')
abline(mod,lty=2,col='darkorange',lwd=1.5)
axis(4,at=c(10,100,150,200))
mtext(4,line=2.5,text = expression(mu),las=2)


tseq=seq(-1800,-700,1)
contemp=numeric(length=length(tseq))
for (t in 1:length(tseq))
{
  contemp[t] = sum(plotSUB$StartBCE < tseq[t]& plotSUB$EndBCE > tseq[t])
}

plot(tseq,contemp,type='l',axes=FALSE,ylim=c(0,40),xlim=c(-1800,-700),
     ylab='N Occupied sites')
axis(side=2)
```

```
par(mar=c(4,4,3,4))
bseq=seq(-1800,-600,100)
freq=table(cut(plotSUB$MidYear,breaks = bseq))
plot(0,0,type='n',axes=FALSE,ylim=c(0,max(freq)),xlim=c(-1800,-700),
     ylab='Frequency of MidPoints')
for (i in 2:(length(bseq)-1))
{
  rect(xleft=bseq[i-1],xright=bseq[i],ybottom=0,
       ytop=freq[i],border=NA,col='lightblue')
}
axis(2)
axis(1,at=seq(-1800,-600,200),labels=seq(1800,600,-200))
```

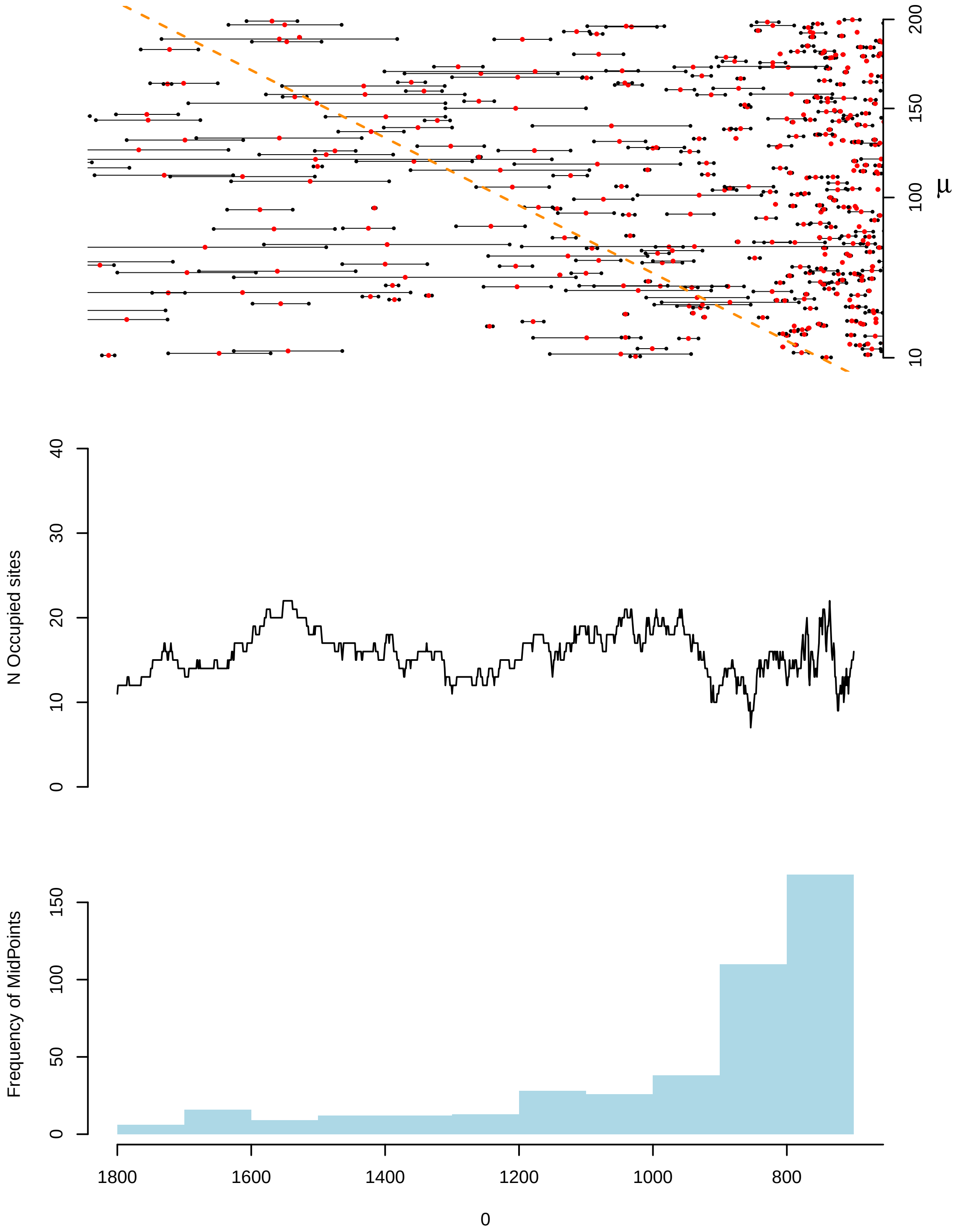

Ignoring duration and treating each site occupation as a point (rather than a line) in time creates a false impression of a increase in the number of sites. The figure below further demonstrates this point by comparing the true density of site frequency (dashed red line) against an envelope of density lines of midpoint frequency across the full 100 simulations.

```r
plot(simyears, mediankd, type="l",  xlim=c(-1750,-751),
     xlab="Years BC", ylab="",axes=FALSE)
axis(1,at=seq(-1600,-800,+200),labels=seq(1600,800,-200))
axis(2)
box()

for (d in 1:ncol(simmat)){
  lines(simyears,simmat[,d], col=rgb(191,191,191,alpha=75,max=255))
}
abline(h=1/length(simyears), col="red", lty="dashed")
lines(simyears,mediankd, col="black")
legend("topleft",
       legend=c("actual mine density", "simulated densities","median simulated"),
       col=c("red", "grey75","black"), lwd=c(1,1,1),
       lty=c("dashed","solid","solid"), bty="n", cex=1)
```

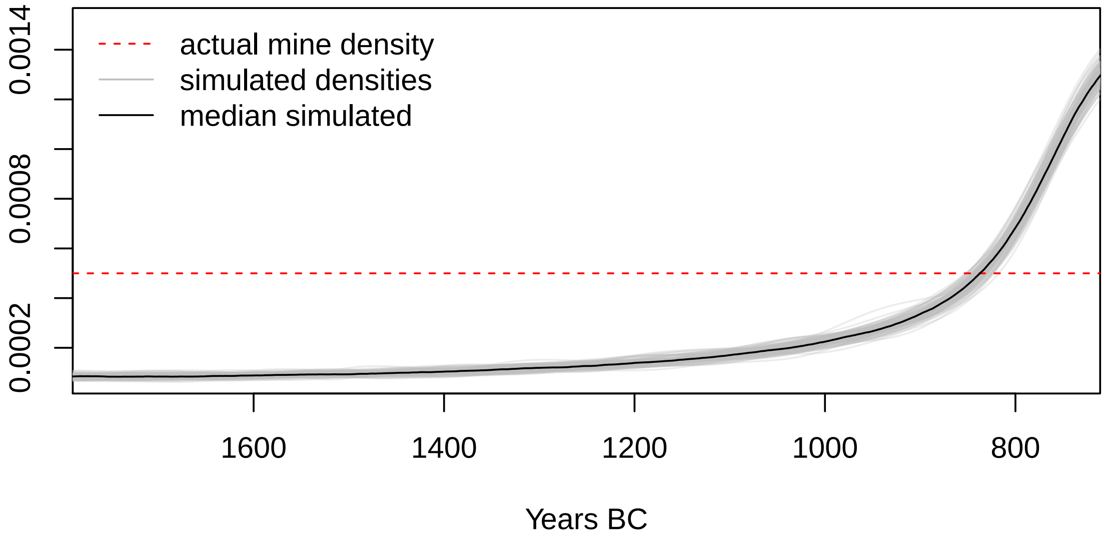

Simulation 3: Nucleation/Dispersal Bias

Setup

Consider two hypothethical archaeological periods, α and β, with equal durations in time. Our objective is to investigate the percentage change in the number of residential units across the two periods. More formally we are intested in estimating $100 \times (N_\beta - N_\alpha)/N_\beta$ where N_α and N_β are the total number of residential units for each period. Our residential units are however spatially orgaised into sites (i.e. settlements) with different sizes, and that our sampling is conditioned by such structure. More specifically, we assume that sampling occurs at the level of site and not each individual residential unit, and that we are able to recover only a fraction r of sites, where $r = k/K$, where k is observed number of sites across the two periods in our sample and K is the number of sites across the two periods in the population. Finally, we assume that the

probability of each site being sampled is defined by the following equation:

$$\pi_i = \frac{S_i^b}{\sum_{j=1}^{K} S_j^b}$$

where π_i is the probability of selecting a site with size S_i, K is the total number of sites, and $0 \leq b \leq 1$. The exponent b is bias parameter that conditions the probability of a site to be sampled as a function of its size. When $b = 0$, all sites have the same chance of being included in the sample, but when $b > 0$ larger sites have a higher probability of being selected.

The figure below shows the impact of different values of b with a hypothethical dataset with $K = 6$ and site sizes $S_1 = 200$, $S_2 = 100$, $S_3 = 50$, $S_4 = 20$, $S_5 = 20$, and $S_6 = 10$.

```
par(mar=c(5,4,3,4))
S=c(200,100,50,20,20,10)
b=c(0,0.2,0.5)
barplot(S,names.arg=c(1:6),col="grey",width=1,space=0.2,border=NA,xlab="Sites")
mtext(side=2,line=3,"S",las=2)
par(new=T)
plot(x=seq(from=0.5/7,by=1.2/7,length.out =6),
     y=S^b[1]/sum(S^b[1]),type='b',pch=20,col='orange',
     axes=F,xlab="",ylab="",xlim=c(0,1),ylim=c(0,0.4))
lines(x=seq(from=0.5/7,by=1.2/7,length.out = 6),
      y=S^b[2]/sum(S^b[2]),type='b',pch=20,col='red')
lines(x=seq(from=0.5/7,by=1.2/7,length.out = 6),
      y=S^b[3]/sum(S^b[3]),type='b',pch=20,col='darkred')
legend("topright",legend=c("b=0","b=0.2","b=0.5"),
       pch=20,lwd=1,col=c('orange','red','red'),bty='n')
axis(side=4,at=c(0,0.1,0.2,0.3,0.4))
mtext(side=4,line=3,expression(pi),las=2)
```

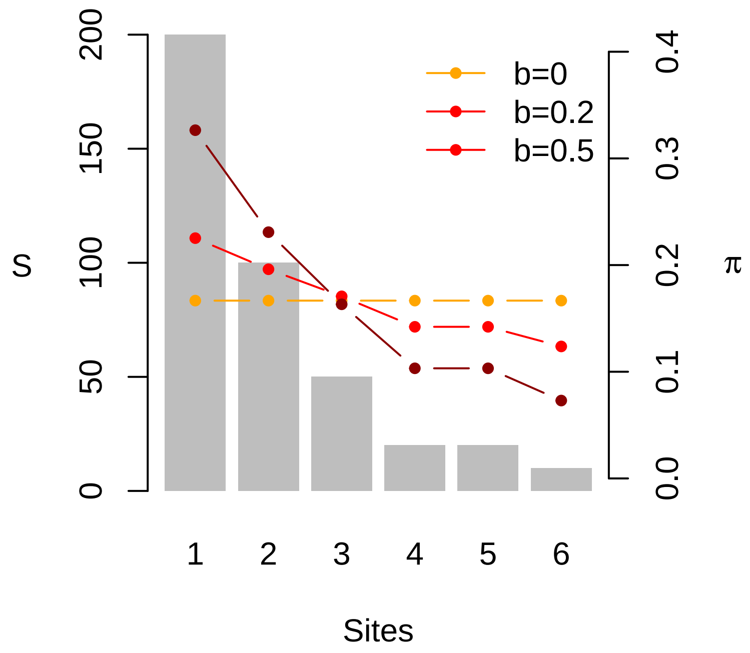

Simulation Experiment

What is the combined impact of the non-random sampling regime described above when the two periods α and β are characterised by a *different* settlement size distribution which we might expect in case of nucleation/dispersal shifts? Here we employ a simple tactical simulation where we: 1) generate artificial settlements for two hypothethical archaeological periods; 2) sample a fraction r of settlements using different degrees of site-size bias b; and 3) compute the observed percentage change in the number of residential units. For period α the site size distribution would be approximately log-normal with $\mu = 3$ and $\sigma = 1$ whilst for period β the size distribution would be approximately uniform. The function `sim.settlement()` will generate the artificial settlements ensuring that the total number of residential units for the two periods are the same.

For example:

```
set.seed(224)
result=sim.settlement(K1=300,K2=300,mu=3,sigma=1)
par(mfrow=c(1,2))
hist(result$t1,xlab="Settlement Size",main=expression(paste("period ", alpha)),col='lightblue')
hist(result$t2,xlab="Settlement Size",main=expression(paste("period ", beta)),col='lightblue')
```

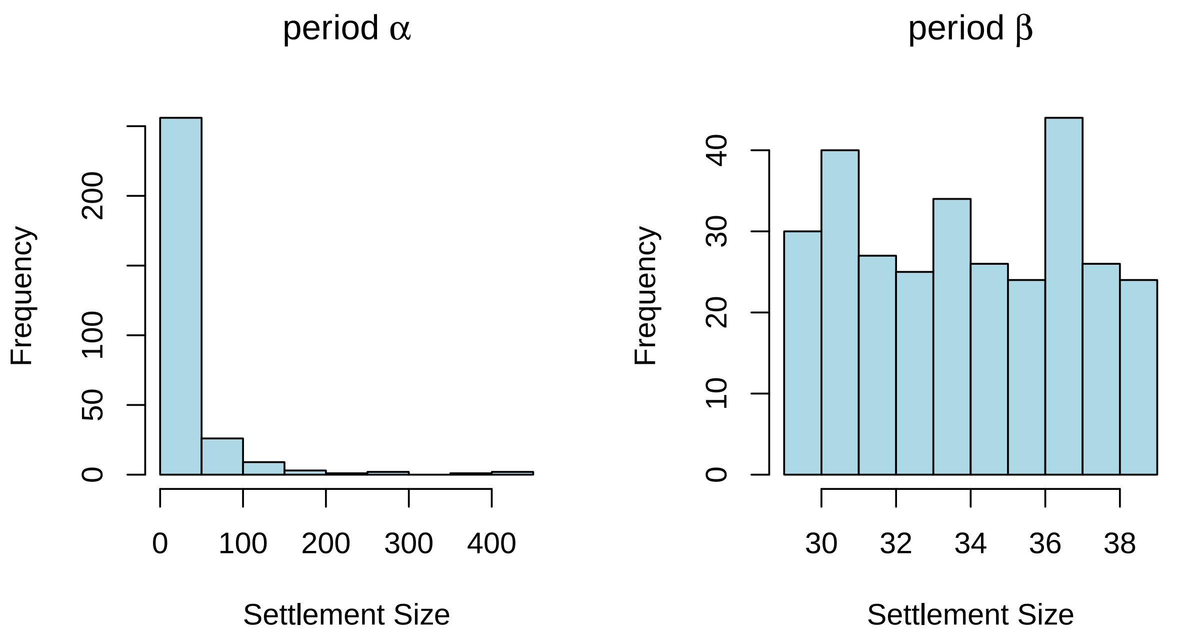


```
# Total number of residential units for each period
sum(result$t1)
```

```
## [1] 10307
```

```
sum(result$t2)
```

```
## [1] 10307
```

To consider different scenarios we consider sampling fractions $r = \{0.1, 0.3, 0.7\}$ and sampling biases $b = \{0, 0.3, 0.7\}$. In all case we consider $K1 = K2 = 1000$ and run 1,000 repetitions for each of the nine parameter combinations.

```r
set.seed(123)
simdata = sim.settlement(K1=1000,K2=1000)
nsim = 100
b = c(0,0.3,0.7)
r = c(0.1,0.3,0.7)
params=expand.grid(r=r,b=b,nsim=1:nsim)
params$pr=NA

for (i in 1:nrow(params))
{
  params$pr[i]=biasedsampling(simdata,r=params$r[i],b=params$b[i])
}

# Plot results
plot(0,0,type='n',xlab=c("b (Sampling Bias)"),
     ylab="Observed Percentage Change",xlim=c(0.5,3.5),
     ylim=range(params$pr),axes=FALSE)

colSeq=c("darkblue","darkorange","darkgrey")
alpha=0.2
colSeq2=c(rgb(0,0,0.54,alpha),rgb(1,0.55,0,alpha),rgb(0.66,0.66,0.66,alpha))
mids=c(0.75,1,1.25)
for (i in 1:length(b))
{
  for (j in 1:length(r))
  {
    bb=b[i]
    rr=r[j]
    y = subset(params,b==bb&r==rr)$pr
    points(x=i-1+runif(100,mids[j]-0.05,mids[j]+0.05),y=y,pch=20,col=colSeq2[j])
    rect(ybottom=quantile(y,0.25),ytop=quantile(y,0.75),
         xleft=i-1+mids[j]-0.07,xright=i-1+mids[j]+0.07,border=colSeq[j])
    lines(x=c(i-1+mids[j]-0.07,i-1+mids[j]+0.07),
          y=c(median(y),median(y)),lwd=2,col=colSeq[j])
  }
}

axis(1,at=c(-2,1,2,3,4),labels=c(NA,0,0.3,0.7,NA))
axis(2)
abline(h=0,lty=2,lwd=1)
text(x=3,y=3,labels="True Percentage Change",cex=0.8)
legend("topright",bty='n',legend=c("r=0.1","r=0.3","r=0.7"),
       col=c("darkblue","darkorange","darkgrey"),
       pch=20,title = "Sampling Fraction")
```

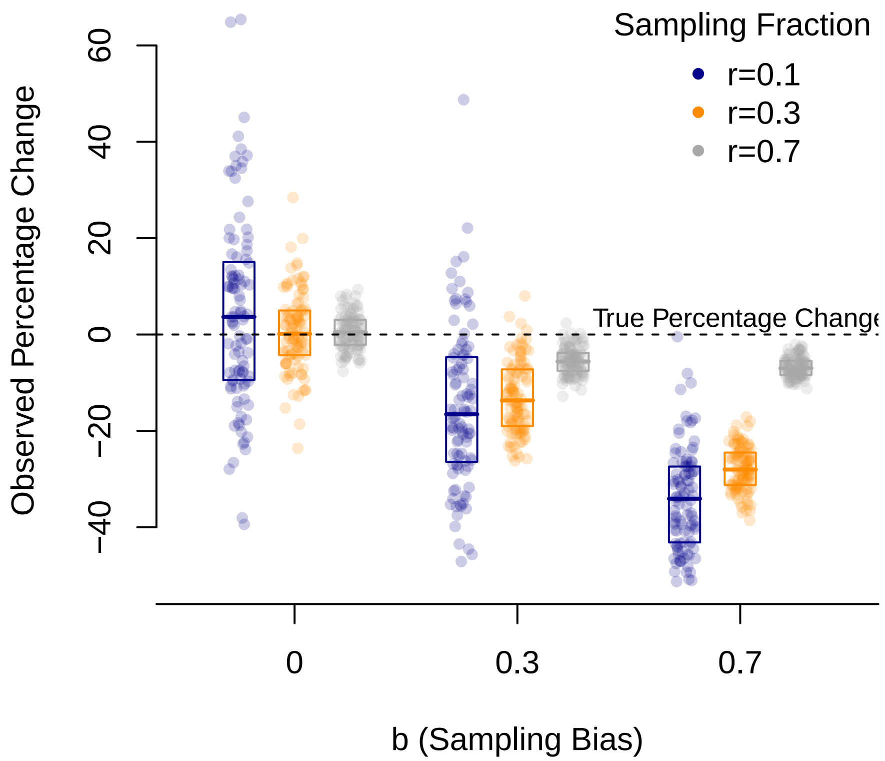
Sampling Fraction
r=0.1
r=0.3
r=0.7
True Percentage Change
Observed Percentage Change
60
40
20
0
−20
−40
0
0.3
0.7
b (Sampling Bias)
